# Rapid Identification of Emerging Human-Pathogenic *Sporothrix* Species with Rolling Circle Amplification

**DOI:** 10.3389/fmicb.2015.01385

**Published:** 2015-12-08

**Authors:** Anderson M. Rodrigues, Mohammad J. Najafzadeh, G. Sybren de Hoog, Zoilo P. de Camargo

**Affiliations:** ^1^Cell Biology Division, Department of Microbiology, Immunology and Parasitology, Federal University of São PauloSão Paulo, Brazil; ^2^Department of Parasitology and Mycology, Ghaem Hospital, School of Medicine, Mashhad University of Medical SciencesMashhad, Iran; ^3^Centraalbureau voor Schimmelcultures, CBS-KNAW Fungal Biodiversity CentreUtrecht, Netherlands

**Keywords:** sporotrichosis, diagnostics, PCR, epidemiology, *Sporothrix*, calmodulin, RCA, identification

## Abstract

*Sporothrix* infections are emerging as an important human and animal threat among otherwise healthy patients, especially in Brazil and China. Correct identification of sporotrichosis agents is beneficial for epidemiological surveillance, enabling implementation of adequate public-health policies and guiding antifungal therapy. In areas of limited resources where sporotrichosis is endemic, high-throughput detection methods that are specific and sensitive are preferred over phenotypic methods that usually result in misidentification of closely related *Sporothrix* species. We sought to establish rolling circle amplification (RCA) as a low-cost screening tool for species-specific identification of human-pathogenic *Sporothrix*. We developed six species-specific padlock probes targeting polymorphisms in the gene encoding calmodulin. BLAST-searches revealed candidate probes that were conserved intraspecifically; no significant homology with sequences from humans, mice, plants or microorganisms outside members of *Sporothrix* were found. The accuracy of our RCA-based assay was demonstrated through the specificity of probe-template binding to 25 *S. brasiliensis*, 58 *S. schenckii*, 5 *S. globosa*, 1 *S. luriei*, 4 *S. mexicana*, and 3 *S. pallida* samples. No cross reactivity between closely related species was evident *in vitro*, and padlock probes yielded 100% specificity and sensitivity down to 3 × 10^6^ copies of the target sequence. RCA-based speciation matched identifications via phylogenetic analysis of the gene encoding calmodulin and the rDNA operon (*kappa* 1.0; 95% confidence interval 1.0-1.0), supporting its use as a reliable alternative to DNA sequencing. This method is a powerful tool for rapid identification and specific detection of medically relevant *Sporothrix*, and due to its robustness has potential for ecological studies.

## Introduction

Sporotrichosis is a chronic fungal infection of humans and animals and is one of the most prevalent (sub)cutaneous mycoses in temperate and subtropical regions of the globe (Chakrabarti et al., 2015). The most probable route of acquisition of the disease is through traumatic introduction of *Sporothrix* propagules into the tissue of the warm-blooded host (De Hoog et al., 2000). The eco-epidemiology of *Sporothrix* species is exceptional in the fungal kingdom due to their frequent epidemic manifestation as large sapronoses or zoonoses (Rodrigues et al., 2013b). The classical environmental route of transmission, which was described more than a century ago, involves traumatic implantation of *Sporothrix* into human skin, usually via contaminated soil or plant material (Zhang et al., 2015). In the alternative route, direct horizontal animal transmission of *Sporothrix* occurs among cats and can lead to zoonotic transmission (cat-to-human), usually via deep scratches and bites from diseased cats (Rodrigues et al., 2014d; Gremião et al., 2015).

Technical developments in investigating the taxonomy of the *S. schenckii* clade have enabled the routine clinical recognition of *S. brasiliensis, S. schenckii s. str., S. globosa*, and *S. luriei* (Marimon et al., 2006, 2007, 2008a). These taxonomical improvements were essential for uncovering important aspects related to species distribution and population structure (Rodrigues et al., 2013b, 2014d), different levels of virulence (Fernandes et al., 2013), host-parasite interplay (Rodrigues et al., 2015c), and varying sensitivities to antifungals (Rodrigues et al., 2014c). The emergence of sporotrichosis in areas where the number of cases remained near baseline for long periods has highlighted the threat of cross-species pathogen transmission, for example of the cat-borne, highly pathogenic clonal offshoot *S. brasiliensis* (Rodrigues et al., 2013b, 2014d, 2015c). On the other hand, the cosmopolitan *S. globosa* is often recovered from human cases after contamination during agricultural practice in Asia (Zhou et al., 2014; Zhang et al., 2015). Different ecological behaviors resulting in distinct routes of contamination underline the importance of strain typing, yet also highlight the need for well-conducted epidemiological investigations to better understand the dynamics and the source of these pathogens in nature.

*Sporothrix brasiliensis* is by far the most virulent species in the clinical clade (Arrillaga-Moncrieff et al., 2009; Fernandes et al., 2013) and is usually related to atypical and more severe clinical manifestation in humans and animals (Almeida-Paes et al., 2014). Recent studies indicated a great proportion of itraconazole-resistant isolates of *S. brasiliensis* during outbreaks. Prolonged exposure to the drugs associated with the clonal population structure during epidemics may be related to the emergence of drug insensitive S*. brasiliensis* isolates. These isolates are continuously spread throughout direct horizontal animal transmission and via zoonotic transmission (Rodrigues et al., 2014c; Borba-Santos et al., 2015; Teixeira et al., 2015). On the other hand, *S. schenckii* presents with a great genetic diversity what is reflected in distinct virulence profiles from low to highly pathogenic genotypes (Arrillaga-Moncrieff et al., 2009; Fernandes et al., 2013) as well it shows a broad *in vitro* susceptibility to azoles (Rodrigues et al., 2014c). Species embedded in the environmental clade such as *S. pallida, S. mexicana*, and *S. chilensis* lack pathogenicity to mammals (Arrillaga-Moncrieff et al., 2009; Rodrigues et al., 2015a) and are usually tolerant to most of the antifungal drugs including azoles (Marimon et al., 2008b; Rodrigues et al., 2014c). Species identification may therefore guide specific treatment, improve our ability to adjust therapeutic regimens and reduce relapse.

Clinically relevant and environmental *Sporothrix* species are endowed with a remarkable phenotypic plasticity, discouraging the use of morphology alone for species identification. This limitation is especially true for agents in the *S. schenckii* clade (*S. brasiliensis, S. schenckii, S. globosa*, and *S. luriei*) (Camacho et al., 2015; Rodrigues et al., 2015a). Uncertainties resulting from classical methods, e.g., assimilation of carbons sources, growth temperature, and micromorphology, highlight the urgent need for development of sensitive and accurate molecular methods for identifying these agents.

The gold standard for identifying species in the *S. schenckii* clade is based on phylogenetic analysis of protein-coding loci, e.g., calmodulin (Marimon et al., 2007), beta-tubulin (Rodrigues et al., 2015a), and translation elongation factor (Rodrigues et al., 2013b; Zhang et al., 2015), as well as the rRNA operon (Zhou et al., 2014). In an epidemic scenario in which hundreds to thousands of cases emerge, as in the long-lasting outbreaks of cat-transmitted sporotrichosis in southeastern Brazil (Rodrigues et al., 2013b) or during the large sapronosis in northeast China (Song et al., 2013; Zhang et al., 2015), DNA sequencing is financially unfeasible for processing a large number of samples. Currently, the major methods available for genotyping and identifying *Sporothrix* down to species level in the clinical laboratory include random amplified polymorphic DNA (Mesa-Arango et al., 2002), amplified fragment length polymorphisms (Zhang et al., 2015), PCR-restriction fragment length polymorphisms (RFLPs) (Rodrigues et al., 2014b), species-specific PCR (Rodrigues et al., 2015b), and protein fingerprinting, for example via matrix-assisted laser desorption/ionization time-of-flight (Oliveira et al., 2015). These methods are routinely implemented as the first means of identification. Limitations may include the need for isolated cultures, highly pure DNA preparations, and/or advanced instruments.

Methods based on the selective amplification and detection of very small quantities of nucleic acids are desirable for diagnosing sporotrichosis (Rodrigues et al., 2015b). Rolling circle amplification (RCA) was first introduced in the 1990s (Fire and Xu, 1995) as a simple and powerful technique for synthesizing large amounts of DNA from low starting concentrations. The method is particularly useful for signal amplification of padlock probes, linear DNA probes that become circularized upon recognition of a specific nucleic-acid sequence. The combination of padlock-probe circularization and amplification through RCA has proven useful for sensitive and specific detection of nucleic-acid sequences from pathogens (Zhang et al., 1998). RCA has gained much popularity in the identification of viral (Haible et al., 2006) and bacterial pathogens (Chen et al., 2014). Despite its limited use in mycological diagnosis, several studies have demonstrated the applicability of RCA for the molecular identification of medically relevant fungi such as *Candida* (Zhou et al., 2008), *Aspergillus* (Zhou et al., 2008), *Trichophyton* (Kong et al., 2008), *Fonsecaea* (Najafzadeh et al., 2011), *Madurella* (Ahmed et al., 2014), and Mucorales (Dolatabadi et al., 2014).

The aim of the present study was to establish a robust screening RCA-based assay for species-specific identification of *S. brasiliensis, S. schenckii s. str., S. globosa, S. luriei, S. mexicana*, and *S. pallida*. We describe the development, optimization, and validation of six species-specific padlock probes for identifying and detecting *Sporothrix* DNA. Improvements in RCA conditions revealed that under isothermal amplification, this method is robust, sensitive, and specific to a diverse panel of clinical and environmental *Sporothrix* strains.

## Materials and methods

### *Sporothrix* strains and culture conditions

A total of 96 reference *Sporothrix* strains obtained from the Federal University of São Paulo, São Paulo, Brazil were used for RCA testing, including clinical and environmental isolates (Table 1). These isolates were previously characterized down to the species level via phylogenetic analysis of the calmodulin-encoding gene (*CAL*) and the rDNA operon (ITS1-5.8S-ITS2), as described elsewhere (Madrid et al., 2010; Silva-Vergara et al., 2012; Rodrigues et al., 2013a,b, 2014b,d; Sasaki et al., 2014). Isolates originated mainly from different regions of Latin America and covered a broad range haplotypes (Rodrigues et al., 2014d), drug sensitivities (Rodrigues et al., 2014c), and clinical manifestations, including the fixed, lymphocutaneous, and disseminated forms of sporotrichosis (Rodrigues et al., 2014d). Cultures were stored as slants on Sabouraud Dextrose agar (Difco Laboratories, Detroit, MI, USA) at room temperature. Type strains representing the main species were included in all experiments. Ethical approval was obtained from the Institutional Ethics in Research Committee (Federal University of São Paulo) under protocol number 0244/11.

**Table 1:** **Strains, species, origin, and GenBank accession numbers of ***Sporothrix*** species used in this study**.

**Isolate code**	**CBS code**	**Species**	**Source**	**Origin**	**GenBank**	
					***CAL***	**ITS**
Ss05	CBS 132985	*S. brasiliensis*	Feline	Brazil	KC693830	KF961142
Ss07	CBS 132986	*S. brasiliensis*	Human	Brazil	KC693831	KF961143
Ss12	–	*S. brasiliensis*	Human	Brazil	KC693835	KF961144
Ss14	–	*S. brasiliensis*	Human	Brazil	KF943632	KF961145
Ss33	–	*S. brasiliensis*	Human	Brazil	KF943637	KF961146
Ss34	–	*S. brasiliensis*	Human	Brazil	KF943638	KF961147
Ss37	–	*S. brasiliensis*	Human	Brazil	KF943639	KF961148
Ss38	–	*S. brasiliensis*	Human	Brazil	KC693844	KF961149
Ss43	–	*S. brasiliensis*	Human	Brazil	JX077112	KF961150
Ss44	–	*S. brasiliensis*	Human	Brazil	KF943641	KF961151
Ss52	–	*S. brasiliensis*	Human	Brazil	KC693845	KF574444
Ss54	CBS 132990	*S. brasiliensis*	Feline	Brazil	JQ041903	JN885580
Ss55	–	*S. brasiliensis*	Human	Brazil	KC693847	KF961152
Ss56	–	*S. brasiliensis*	Human	Brazil	KC693848	KF961153
Ss57	–	*S. brasiliensis*	Human	Brazil	KF943645	KF961154
Ss62	CBS 132991	*S. brasiliensis*	Human	Brazil	JX077113	KF961120
Ss69	–	*S. brasiliensis*	Human	Brazil	KC693849	KF961122
Ss82	CBS 132992	*S. brasiliensis*	Human	Brazil	KC693857	KF961125
Ss87	CBS 132993	*S. brasiliensis*	Human	Brazil	KC693858	KF961126
Ss99	–	*S. brasiliensis*	Human	Brazil	KF574460	KF574442
Ss104	–	*S. brasiliensis*	Human	Brazil	KF574461	KF574443
Ss128	–	*S. brasiliensis*	Human	Brazil	KC693861	KF961155
Ss265	CBS 133020	*S. brasiliensis*	Human	Brazil	JN204360	KF574445
ATCC 4823^G^	CBS 132021	*S. brasiliensis*	Feline	Brazil	KF574459	JQ070114
IPEC16490^T^	CBS 120339^T^	*S. brasiliensis*	Human	Brazil	AM116899	KF574440
CBS 101570	CBS 101570	*S. brunneoviolacea*	Endophyte	USA	KP017101	KC113235
Ss469^T^	CBS 139891	*S. chilensis*	Human	Chile	KP711815	KP711811
Ss470	CBS 139890	*S. chilensis*	Soil	Chile	KP711816	KP711812
Ss06	CBS 132922	*S. globosa*	Human	Brazil	JF811336	JN885574
Ss41	CBS 132923	*S. globosa*	Human	Brazil	JF811337	KF574456
Ss49	CBS 132924	*S. globosa*	Human	Brazil	JF811338	KF961180
Ss236	CBS 132925	*S. globosa*	Human	Brazil	KC693877	KF961181
FMR 8600^T^	CBS 120340	*S. globosa*	Human	Spain	AM116908	FN549905
FMR 9280^T^	CBS 937.72	*S. luriei*	Human	South Africa	AM747302	AB128012
Ss132	CBS 132927	*S. mexicana*	Human	Brazil	JF811340	KF574457
Ss133	CBS 132928	*S. mexicana*	Human	Brazil	JF811341	KF961182
FMR 9107	CBS 120342	*S. mexicana*	Vegetal	Mexico	AM398392	–
FMR 9108^LT^	CBS 120341	*S. mexicana*	Soil	Mexico	AM398393	FN549906
FMR 8939^T^	CBS 302.73	*S. pallida*	Soil	UK	AM398396	–
Ss327	–	*S. pallida*	Feline	Brazil	KF574471	KF574458
CBS 111110	CBS 111110	*S. pallida*	Insect	Germany	AM398382	–
Ss03	CBS 132963	*S. schenckii*	Human	Brazil	JX077117	KF574446
Ss04	–	*S. schenckii*	Human	Brazil	JX077118	KF961156
Ss13	–	*S. schenckii*	Human	Brazil	KC693836	KF961157
Ss15	–	*S. schenckii*	Human	Brazil	KC693837	KF961158
Ss16	–	*S. schenckii*	Human	Brazil	JQ041898	KF961159
Ss17	–	*S. schenckii*	Human	Brazil	KC693838	KF961160
Ss19	–	*S. schenckii*	Human	Brazil	KF943633	KF961161
Ss22	CBS 132964	*S. schenckii*	Human	Brazil	KF943634	KF961162
Ss36	–	*S. schenckii*	Human	Brazil	KC693843	KF961163
Ss39	–	*S. schenckii*	Human	Brazil	JQ041899	JN885576
Ss40	–	*S. schenckii*	Human	Brazil	JQ041900	JN885577
Ss42	CBS 132966	*S. schenckii*	Human	Brazil	KF943640	KF961164
Ss45	–	*S. schenckii*	Human	Brazil	KJ020358	KF961165
Ss46	–	*S. schenckii*	Human	Brazil	KF943642	KF961166
Ss47	–	*S. schenckii*	Human	Brazil	JQ041901	KF961118
Ss48	–	*S. schenckii*	Human	Brazil	KF943643	KF961167
Ss58	–	*S. schenckii*	Human	Brazil	KF943646	KF961119
Ss59	–	*S. schenckii*	Human	Brazil	KF943647	KF961168
Ss61	–	*S. schenckii*	Soil	Brazil	KF561244	KF574447
Ss63	CBS 132968	*S. schenckii*	Human	Brazil	JX077123	KF961121
Ss64	–	*S. schenckii*	Human	Brazil	JX077124	KF961169
Ss73	–	*S. schenckii*	Human	Brazil	KC693853	KF961170
Ss75	–	*S. schenckii*	Human	Brazil	KC693854	KF961123
Ss78	–	*S. schenckii*	Human	Brazil	KC693855	KF961171
Ss80	CBS 132969	*S. schenckii*	Human	Brazil	JX077125	KF961124
Ss102	CBS 132970	*S. schenckii*	Human	Brazil	KF943671	KF961172
Ss105	–	*S. schenckii*	Human	Brazil	KF943673	KF961127
Ss107	–	*S. schenckii*	Human	Brazil	KF943675	KF961128
Ss109	–	*S. schenckii*	Human	Brazil	KF943676	KF961129
Ss110	–	*S. schenckii*	Human	Brazil	KF943677	KF961173
Ss113	CBS 132972	*S. schenckii*	Human	Brazil	KF943679	KF961130
Ss116	–	*S. schenckii*	Human	Brazil	KF943680	KF961131
Ss118	CBS 132974	*S. schenckii*	Human	Brazil	JX077126	KF961174
Ss119	–	*S. schenckii*	Human	Brazil	KF943682	KF961175
Ss122	–	*S. schenckii*	Human	Brazil	KF943685	KF961132
Ss123	–	*S. schenckii*	Human	Brazil	KF943686	KF961133
Ss124	–	*S. schenckii*	Human	Brazil	KF943687	KF961176
Ss126	–	*S. schenckii*	Human	Brazil	JQ041904	JN885581
Ss129	–	*S. schenckii*	Human	Brazil	KF943689	KF961134
Ss130	–	*S. schenckii*	Human	Brazil	KF943690	KF961135
Ss136	–	*S. schenckii*	Human	Brazil	KF943692	KF961177
Ss137	–	*S. schenckii*	Human	Brazil	KF574462	KF574448
Ss138	–	*S. schenckii*	Human	Brazil	KF943693	KF961136
Ss140	–	*S. schenckii*	Human	Brazil	KF574463	KF574449
Ss141	CBS 132975	*S. schenckii*	Human	Brazil	JQ041905	JN885582
Ss143	–	*S. schenckii*	Human	Brazil	JQ041906	JN885583
Ss144	–	*S. schenckii*	Human	Brazil	KF943695	KF961178
Ss158	–	*S. schenckii*	Human	Brazil	KF943698	KF961137
Ss190	–	*S. schenckii*	Human	Brazil	KF943701	KF961138
Ss240	–	*S. schenckii*	Human	Brazil	KF943704	KF961141
Ss159	CBS 132976	*S. schenckii*	Human	Japan	KF574464	KF574450
Ss160	–	*S. schenckii*	Human	Mexico	KF574465	KF574451
Ss161	–	*S. schenckii*	Human	Mexico	KF574466	KF574452
Ss162	CBS 132977	*S. schenckii*	Vegetal	Mexico	KF574467	KF574453
Ss163	–	*S. schenckii*	Human	Peru	KF574468	KF574454
Ss164	–	*S. schenckii*	Human	Peru	KF574469	KF574455
ATCC 4821^G^	CBS 132984	*S. schenckii*	Human	USA	KF574470	JQ070112
CBS 359.36^T^	CBS 359.36	*S. schenckii*	Human	USA	AM117437	FJ545232
FMR 9338^T^	CBS 124561	*S. brunneoviolacea*	Soil	Spain	KF574472	FN546959
FMR 8977	CBS 125442	*S. dimorphospora*	Soil	Spain	FN546961	–
C8213	–	*Ophiostoma stenoceras*	Human	Venezuela	KF478913	KJ999893
Pb18	–	*Paracoccidioides brasiliensis*	Human	Brazil	–	–
832	–	*Histoplasma capsulatum*	Human	Brazil	–	–
CBS 2530	CBS 2530	*Trichosporon asahii*	Human	Brazil	–	–
ATCC 10231	CBS 6431	*Candida albicans*	Human	Unknown	–	–
CBS 10079	CBS 10079	*Cryptococcus neoformans*	Human	Australia	–	–

*IPEC, Instituto de Pesquisa Clínica Evandro Chagas, Fiocruz, Brazil; FMR, Facultat de Medicina i Ciències de la Salut, Reus, Spain; CBS, Centraalbureau voor Schimmelcultures, Utrecht, The Netherlands; ATCC, American Type Culture Collection, Manassas, USA; NK, not known*;

T *, type strain*;

G *, genome. All “Ss” strains belong to the culture collection of Federal University of SP (UNIFESP)*.

### DNA extraction

DNA was extracted and purified directly from fungal colonies with the FastDNA Kit (MP Biomedicals, Vista, CA, USA), in accordance with the manufacturer's instructions. Briefly, 10-day old colonies were transferred to screw-cap tubes containing ceramic beads 0.25″ in diameter plus matrix A and 1 mL CLS-Y. Suspensions were homogenized three times in a Precellys 24-Dual homogenizer (Bertin Technologies, France) at 6000 rpm for 20 s with 15-s intervals. DNA concentration was determined with a NanoDrop 2000 spectrophotometer (Thermo Fisher Scientific, USA), based on the default value [1 optical density (OD) unit = 50 mg/mL double-stranded DNA]; thereafter, DNA was diluted to a final concentration of 100 ng/μL. DNA quality was evaluated by determining ODs at wavelengths of 260 and 280 nm, and calculating the OD_260∕280_ ratio; only samples with OD_260∕280_ = 1.8−2.0 were used in further analyses. DNA samples were stored at −20°C until use in PCR. The quality of the extracted DNA was assessed by amplification of part of the rDNA operon using the universal primers ITS1 (5′-TCC GTA GGT GAA CCT TGC GG-3′) and ITS4 (5′-TCC TCC GCT TAT TGA TAT GC-3′) (White et al., 1990). Amplified products were separated via agarose gel electrophoresis. Amplification of a single product indicated that the sample was free of PCR inhibitors.

### Primer design and PCRs

*CAL* sequences from 277 isolates belonging to the *S. schenckii* complex, (Rodrigues et al., 2014d) allied species and genera (*Ophiostoma* and *Grosmannia*), and other pathogens were included in order to develop new primers targeting exons 3–5 of *CAL*. These sequences were previously deposited online at GenBank and described by Marimon et al. (2006, 2007, 2008a); Rodrigues et al. (2013a,b, 2014b,d, 2015a), Fernandes et al. (2013), and Sasaki et al. (2014). Nucleotide sequences were aligned using MAFFT v.7 (Katoh and Standley, 2013). The *CAL* alignment was corrected manually using MEGA 6 (Tamura et al., 2013) in order to avoid mispaired bases. Primer3 (http://primer3.wi.mit.edu/) (Untergasser et al., 2012) was used to evaluate melting temperature, %GC content, dimers, and mismatches in candidate sequences. Candidate primer pairs were evaluated with Mfold (Zuker, 2003) for potential secondary structures that would reduce the efficiency of amplification. The specificity of the new *CAL* primer pair was further validated *in silico* with Primer-BLAST (http://www.ncbi.nlm.nih.gov/tools/primer-blast/) (Ye et al., 2012).

*Sporothrix* total DNA was directly used as template in PCRs with the new *CAL* primer pair. Reactions were performed in a final volume of 12.5 μL, including 6.25 μL PCR Master Mix buffer (2 ×; 3 mM MgCl_2_, 400 mM each dNTP, and 50 U/mL Taq polymerase; Promega, Madison, WI, USA), 4.25 μL water, 0.5 μL forward primer *CAL*-Fw (5′-CGC AAT GCC AGG CCG AGT CAC-3′; 10 pmol/μL), 0.5 μL reverse primer *CAL*-Rv (5′-ATT TCT GCA TCA TGA GCT GGA C-3′; 10 pmol/μL), and 1 μL target DNA (100 ng/μL). PCRs were performed in an Eppendorf Mastercycler Pro (Eppendorf, Hamburg, Germany). PCR conditions consisted of an initial step of 95°C for 4 min followed by 35 cycles of 1 min at 94°C, 1 min at 60°C, and 1 min at 72°C, as well as a final extension step of 10 min at 72°C. Aliquots (3 μL) of the PCR products were analyzed on a 1.2% agarose gel (100 V, 60 min) in the presence of GelRed™ (Biotium, Hayward, CA, USA) and photographed under ultraviolet illumination using the L-Pix Touch (Loccus Biotecnologia, São Paulo, Brazil) imaging system (Rodrigues et al., 2015b).

### Phylogenetic analysis

Amplified products were gel-purified with the Wizard SV Gel and PCR Clean-Up System (Promega) in accordance with the manufacturer's instructions. DNA samples were sequenced with an ABI 3730 DNA Analyzer 48-well capillary sequencer (Applied Biosystems, Foster City, CA, USA) using the DYEnamic ET Dye Terminator Kit with Thermo Sequenase II DNA Polymerase (Applied Biosystems). Fragments were sequenced on both strands to increase the quality of sequence data (*Phred* > 30) and assembled into consensus sequences using CAP3 (Hall, 1999). Consensus sequences were used for BLAST.

Genetic relationships were investigated via phylogenetic analysis using neighbor-joining-, maximum likelihood-, and maximum parsimony-based methods. Phylogenetic trees based on a combined dataset (*CAL* + rDNA operon) were constructed in MEGA6 (Tamura et al., 2013). Evolutionary distances were computed using Kimura's two-parameter model (Kimura, 1980), and the robustness of branches was assessed by bootstrap analysis of 1000 replicates (Felsenstein, 1985).

### Padlock probe design

Padlock probes were designed based on the dataset described above. All *CAL* haplotypes previously described by our group (Fernandes et al., 2013; Rodrigues et al., 2013a,b, 2014b,d; Sasaki et al., 2014) were aligned using MAFFT v.7 and screened for informative nucleotide polymorphisms that were conserved within a single species and divergent between species. Type strains were included for all *Sporothrix* species evaluated.

**Figure 1:**
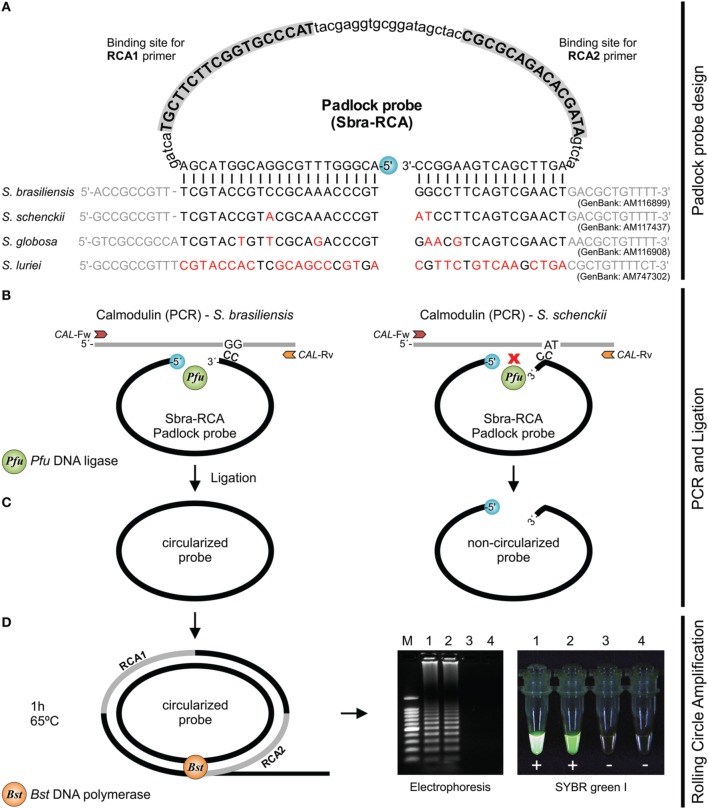
**Schematic of RCA of circularized padlock probes**. **(A)** *S. brasiliensis* (Sbra-RCA) padlock probe design. **(B)** *CAL* is amplified by PCR with primers *CAL*-Fw and *CAL*-Rv; PCR products are submitted to ligation. Circularization of padlock probes occurs only if both probe arms hybridize correctly to the target sequence. **(C)** Upon specific hybridization, the phosphorylated 5′ end and the free hydroxyl at the 3′ end of the probe are joined by *Pfu* DNA ligase. After ligation, non-circularized probes and single-stranded primers are removed with Exo I and Exo III (optional step). **(D)** Circularized padlock probes serve as DNA template and signal amplification occurs via RCA, using *Bst* DNA polymerase and the primers RCA1 and RCA2. DNA synthesis occurs continuously for 1 h under isothermal amplification (65°C). DNA products are detected by gel electrophoresis or directly with SYBR Green I.

Six species-specific padlock probes were designed. Primer3 was used to evaluate melting temperature, %GC content, dimers, and mismatches in candidate padlock probes. To enhance specific binding, the 5′ end probe-binding arm was constructed with minimal secondary structure (evaluated in Mfold) and a melting temperature of 66~70°C. The ligation temperature was set to 63°C. To increase specificity, target-complementary sites were chosen at the 3′ end probe-binding arm with a melting temperature of 48–53°C (Najafzadeh et al., 2011; Lackner et al., 2012). The schematic of the RCA method is shown in Figure 1. Padlock probes were synthesized at Integrated DNA Technologies (USA) using Ultramer DNA Oligo technology at a final scale of 4 nmol. The 5′-terminal end of the padlock probes was phosphorylated (Figure 1). *In silico* specificity of the 5′ and 3′ end probe-binding arms were verified against sequences from *Sporothrix*, including the type strains.

### Ligation

Ligation reactions were performed in a final volume of 10 μL, including 1 μL purified *CAL* amplicon (2–5 pmol/μL) mixed with 0.1 μL *Pfu* DNA ligase (4 U/μL; Agilent Technologies, USA), 2 μL padlock probe (0.1 μM; Integrated DNA Technologies), 1 μL 10 × buffer (20 mM Tris-HCl (pH 7.5), 20 mM KCl, 10 mM MgCl_2_, 0.1% IGEPAL, 0.01 mM rATP, and 1 mM dithiothreitol; Agilent Technologies), and 5.9 μL ultra-pure water. A reaction containing all these components except the *CAL* amplicon served as the negative control. Ligation started with 5 min of pre-denaturation at 94°C, followed by five cycles of 94°C for 30 s and 4 min at 63°C. Normally, RCA can be performed with one denaturation cycle followed by one ligation cycle, but we used five cycles in order to improve the yield of RCA products (Kong et al., 2008).

### Exonucleolysis

Non-circularized padlock probes and excess primers were removed after ligation via endonuclease digestion at 37°C in a final volume of 20 μL. The exonucleolysis mix consisted of 0.5 μL Exo I (New England BioLabs, Ipswich, MA, USA), 1 μL 10 × Exo I Buffer (New England BioLabs), 0.2 μL Exo III (New England BioLabs), 1 μL 10 × Exo III Buffer (New England BioLabs), and 7.3 μL ultra-pure water. The exonucleolysis mix (10 μL) was then directly mixed with 10 μL of the ligation reaction and incubated at 37°C for 30 min. Enzyme activity was stopped via incubation at 94°C for 3 min.

### RCA

RCAs were run in duplicate in total volumes of 25 μL with 3 μL of enzyme-treated ligation product as template. The RCA mix contained 4 U *Bst* DNA polymerase (New England BioLabs), 2.5 μL 10 × *Bst* Thermopol reaction buffer (containing 20 mM Tris-HCl (pH 8.8), 10 mM KCl, 10 mM (NH_4_)_2_SO_4_, and 0.1% Triton X-100; New England BioLabs), 200 μM deoxynucleoside triphosphate mix (Promega), 0.5 μL RCA1 primer (10 μM; Integrated DNA Technologies, USA), and 0.5 μL RCA2 primer (10 μM; Integrated DNA Technologies, USA). RCAs were incubated for 60 min at 65°C. A negative control reaction contained all RCA components except enzyme-treated ligation product as template. Aliquots (10 μL) of the reactions were resolved on 1.2% (w/v) agarose gels for 60 min at 100 V in the presence of GelRed™. We included a lane loaded with GeneRuler 100 bp Plus DNA Ladder (Thermo Fisher Scientific, USA). Bands were visualized using the L-Pix Touch imaging system. For direct visual detection of replicated DNA in the RCA reactions, 1 μL 10-fold diluted (original 10,000 ×) SYBR Green I (Sigma-Aldrich, USA) was added to the reaction tubes and imaged under ultraviolet illumination using the L-Pix Touch imaging system.

### Sensitivity and detection limit

*CAL* amplicons were purified with the Wizard SV Gel and PCR Clean-Up System (Promega) in accordance with the manufacturer's instructions. *CAL* concentration was determined with a NanoDrop 2000 spectrophotometer as described above; thereafter, *CAL* DNA was diluted to a final concentration of 300 ng/μL. Copy numbers were calculated with an online tool based on Avogadro's number (http://cels.uri.edu/gsc/cndna.html; accessed in May 1, 2015). For calculation, an amplicon length of 850 bp was assumed. We evaluated the sensitivity of each padlock probe to ensure reliable amplification at low levels of target DNA. We performed 10-fold serial dilutions of *CAL* DNA, starting with 3 × 10^11^ copies per tube and ending with 3 copies per tube. The detection limit was noted for each padlock probe.

### Specificity of padlock probes

Padlock-probe specificity was determined using *CAL* amplicons from closely related *Sporothrix* species. Each probe was tested *in vitro* against several non-target *CAL* sequences belonging to clinical (*S. brasiliensis, S. schenckii, S. globosa*, and *S. luriei*) and environmental (*S. mexicana* and *S. pallida*) species. In addition, we used several sequences from other medically relevant fungi, including agents of superficial, subcutaneous, and systemic mycosis in humans and animals. RCA conditions and gel electrophoresis were as described above.

### Statistical analysis

To measure the degree of concordance of the results from RCA and phylogeny-based classification (*CAL*+ITS) (Rodrigues et al., 2014d) or RCA and species-specific PCR (Rodrigues et al., 2015b), we calculated the *kappa* statistic and its 95% confidence interval. *Kappa* values were interpreted as follows: 0.00–0.20, poor agreement; 0.21–0.40, fair agreement; 0.41–0.60, moderate agreement; 0.61–0.80, good agreement; 0.81–1.00, very good agreement (Altman, 1991). All calculations were performed with MedCalc Statistical Software version 14.8.1 (MedCalc Software bvba; http://www.medcalc.org).

### Detection of *Sporothrix* DNA from other sources

To demonstrate the feasibility of our RCA approach for sensitive and sequence-specific detection of *Sporothrix* DNA within a complex mixture containing vegetable/host nucleic acids, we used artificially contaminated (spiked) environmental and animal samples. *Sporothrix brasiliensis* (CBS 132990), *S. schenckii* (CBS 359.36), *S. globosa* (CBS 120340), *S. luriei* (CBS 937.72), *S. mexicana* (CBS 120341), and *S. pallida* (CBS 302.73) were cultured on potato dextrose agar plates (Difco Laboratories, Detroit, MI, USA) at room temperature for 7 days to promote conidiation. For environmental detection, a conidial suspension was prepared with sterile saline solution and adjusting the OD 520 nm to 0.2, which approximately corresponds to a concentration of 10^6^ cells mL^−1^. Thereafter, a total of 50 μL of each dilution of the conidial suspension was added to screw cap tubes (as described in *2.2 DNA extraction*) and co-extracted with soil (100 mg), *Sphagnum* moss (50 mg) or rose leaves (*Rosa* spp., 50 mg), corresponding the main sources of *Sporothrix* in nature reported in the literature (Rodrigues et al., 2014a). DNA extraction procedures were the same as described above except that for environmental samples, instead of CLS-Y, we used 800 μL of the CLS-VF plus 200 μL of the PPS solutions from Fast DNA kit (MP Biomedicals, Vista, CA, USA). Negative control included non-spiked environmental samples. As a quality control, all samples (spiked and non-spiked) were also tested by PCR using species-specific primers as described elsewhere (Rodrigues et al., 2015b) to discharge any prior contamination with *Sporothrix* DNA as well as to check the presence of PCR inhibitors, which are often co-extracted with soil or plants. In doing so, we evaluated both, the presence of *Sporothrix* DNA (before spike) and the absence of PCR inhibitors (after spike).

For detection from host samples, we used DNA from non-infected BALB/c mice (Rodrigues et al., 2015b). Briefly, fresh tissue fragments (~100 mg) from the spleen, lungs, tail and feces were placed into screw cap tubes containing a 0.25″ in diameter plus matrix A and 1 mL CLS-TS (MP Biomedicals, Vista, CA, USA). Tissue DNA were extracted as described elsewhere (Rodrigues et al., 2015b). Animal DNA quality was assessed by amplifying the β-actin gene in the BALB/c genome as described by Pahl et al. (1999). Samples that generated positive amplification signals were regarded to be free of PCR inhibitors. DNA extracted from tissue samples (diluted at 100 ng/μl) were spiked with 100 ng of a single *Sporothrix* spp. DNA (10:1). This study was performed in strict accordance with recommendations in the Guide for the Care and Use of Laboratory Animals of the National Institutes of Health.

Afterwards, for both environmental and clinical samples (spiked and non-spiked), 1 μl was taken and directly used as templates in PCR reactions with the primers *CAL*-Fw and *CAL*-Rv as described above (see Section Primer design and PCRs). Thereafter, 1 μL of amplicons were submitted to ligation (see Section Ligation), exonucleolysis (see Section Exonucleolysis), and RCA (see Section RCA). As a positive control we included a reaction containing *CAL* amplicon as a target for each specific probe. A negative control reaction contained all RCA components except enzyme-treated ligation product as template (blank).

## Results

**Figure 2:**
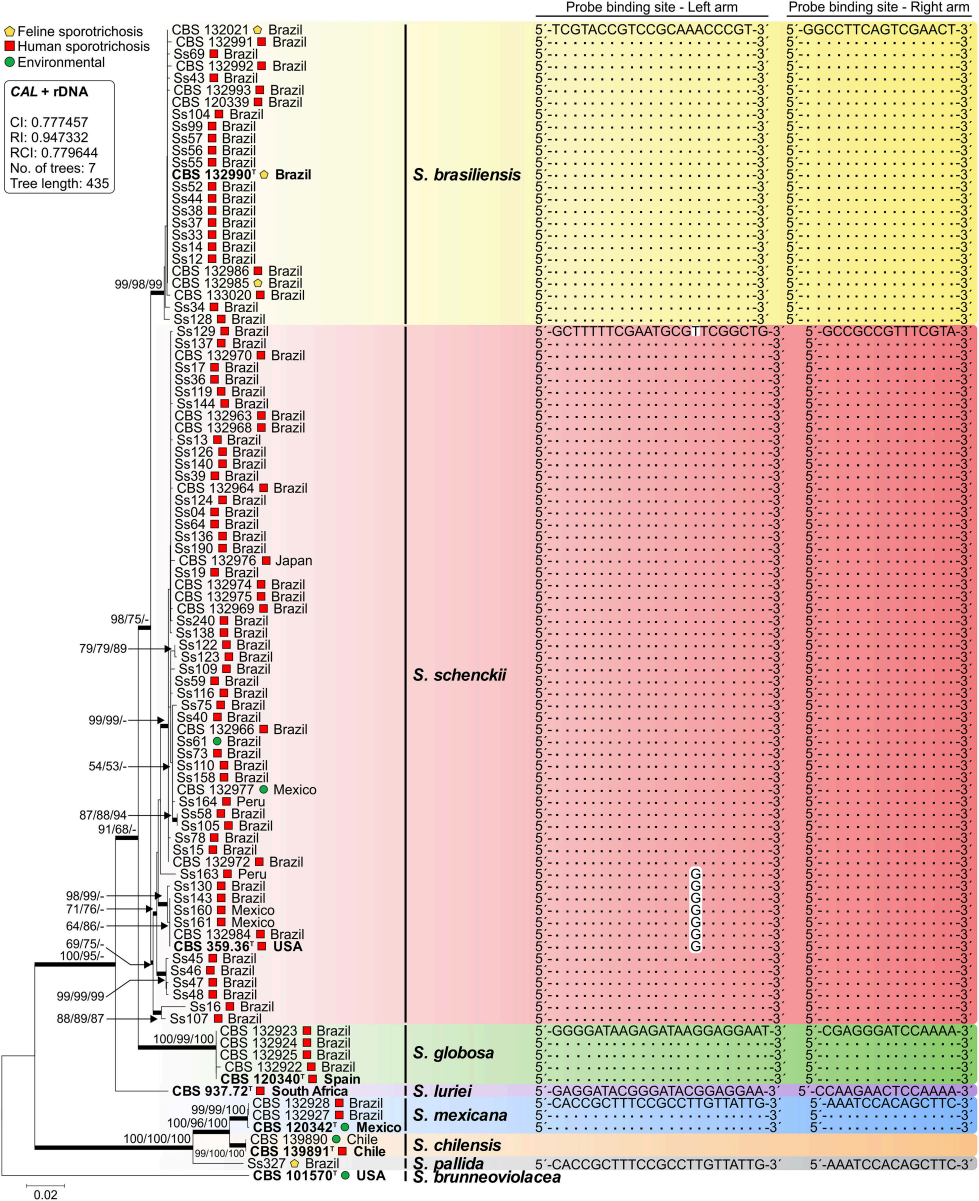
**Phylogenetic tree generated by neighbor joining, maximum likelihood, and maximum parsimony using partial nucleotide sequences of ***CAL*** and the rDNA operon (ITS1-5.8S-ITS2)**. Bootstrap values (1000 replicates) were added (denoted as neighbor joining/maximum likelihood/maximum parsimony). Bar, total nucleotide differences between taxa; ^T^ = type strain. The box contains statistics from maximum parsimony: CI, consistency index; RI, retention index; RCI, composite index (for all sites). Sequence conservation through a genetically diverse panel of *Sporothrix* for both left and right arms of each padlock probe is indicated. Further information about isolate sources appears in Table 1.

Isolates used to develop and validate RCA were previously identified down to the species level via DNA sequencing and phylogenetic analysis of *CAL* and the rDNA ITS region (Rodrigues et al., 2014d). The final dataset had 1359 characters, of which 450 were variable, 280 were parsimony-informative, and 169 were singleton. Sequences from a genetically diverse panel of *Sporothrix* isolates clustered into eight groups with high bootstrap support, matching the species previously described in the literature (Figure 2). *In silico* analyses of the 5′ and 3′ end probe-binding arms revealed that RCA probes were conserved within a single species (Figure 2). In addition, the sequence homology of each *CAL* primer as well as RCA probes sequences were assessed with Primer-BLAST, and no significant homology was found with human, plant, mouse, or microorganism sequences outside the genus of *Sporothrix*.

Compared to other markers (e.g., β-tubulin, translation elongation factor 1α, the rDNA operon, and chitin synthase (Marimon et al., 2006; Zhang et al., 2015), the *CAL* region offers a substantial number of interspecific variations (205 parsimony-informative sites for *CAL* vs. 75 in the rDNA) that supports the use of *CAL* to discriminate closely related *Sporothrix* (Table 1). Such variation led us to choose *CAL* to develop six padlock probes targeting species-specific regions of human-pathogenic *Sporothrix* species (Table 2). Probes were applied *in vitro* during isothermal RCA to identify 25 *S. brasiliensis*, 58 *S. schenckii s. str*., 5 *S. globosa*, 1 *S. luriei*, 4 *S. mexicana*, and 3 *S. pallida* samples. Typically, each padlock probe was hybridized to a *CAL* target (~850 bp) to form a circular probe by *Pfu* DNA ligase when there was a perfect probe-template match. Circularized probes were amplified with replication primers RCA1 and RCA2 with *Bst* DNA polymerase. Amplification was achieved for all species evaluated in the conditions described.

**Table 2:** **Details of species-specific sites and padlock probes targeting ***CAL*** in ***Sporothrix*** spp**.

**Organism target**	**Padlock probe/primer**	**Probes and primers sequences**
*S. brasiliensis*	Sbra-RCA	5′-P-ACGGGTTTGCGGACGGTACGAGatcaTGCTTCTTCGGTGCCCAT tacgaggtgcggatagctacCGCGCAGACACGATAgtctaAGTTCGACTGAAGGCC-3′
*S. schenckii*	Ssch-RCA	5′-P-CAGCCGAMCGCATTCGAAAAAGCGatcaTGCTTCTTCGGTGCCCAT tacgaggtgcggatagctacCGCGCAGACACGATAgtctaTACGAAACGGCGGC-3′
*S. globosa*	Sglo-RCA	5′-P-ATTCCTCCTTATCTCTTATCCCCGatcaTGCTTCTTCGGTGCCCAT tacgaggtgcggatagctacCGCGCAGACACGATAgtctaTTTTGGATCCCTCG-3′
*S. luriei*	Slur-RCA	5′-P-TTCCTCCGTATCCCGTATCCTCGatcaTGCTTCTTCGGTGCCCAT tacgaggtgcggatagctacCGCGCAGACACGATAgtctaTTTTGGAGTTCTTGG-3′
*S. mexicana*	Smex-RCA	5′-P-CAATAACAAGGCGGAAAGCGGTGGatcaTGCTTCTTCGGTGCCCAT tacgaggtgcggatagctacCGCGCAGACACGATAgtctaGAAGCTGTGGATTT-3′
*S. pallida*	Spa-RCA	5′**-**P**-**CGCGAAAATGGCGGAAAGCAGCGatcaTGCTTCTTCGGTGCCCAT tacgaggtgcggatagctacCGCGCAGACACGATAgtctaGAGTTTCCAAGCAC-3′
	RCA1	5′-ATG GGC ACC GAA GAA GCA-3′
	RCA2	5′-CGC GCA GAC ACG ATA-3′
	*CAL*-Fw	5′-CGC AAT GCC AGG CCG AGT CAC-3′
	*CAL*-Rv	5′-ATT TCT GCA TCA TGA GCT GGA C-3′

*5′-P: The 5′ terminal end of padlock probe P was phosphorylated*.

**Figure 3:**
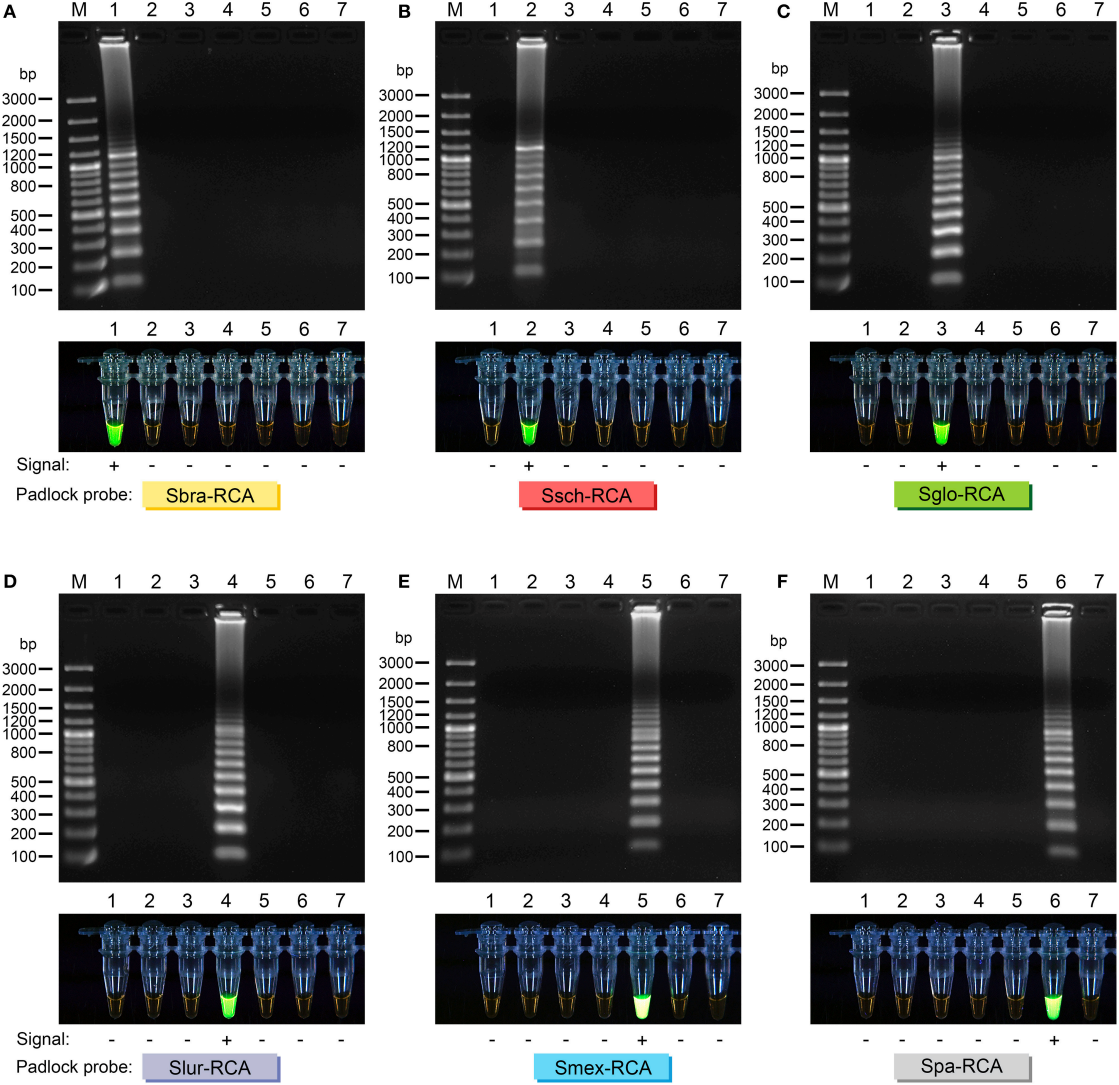
**Agarose gel electrophoresis of successful RCA of (A) Sbra-RCA padlock probe; (B) Ssch-RCA padlock probe; (C) Sglo-RCA padlock probe; (D) Slur-RCA padlock probe; (E) Smex-RCA padlock probe; and (F) Spa-RCA padlock probe**. Gel lanes: 1, *S. brasiliensis* (CBS 132990); 2, *S. schenckii* (CBS 359.36); 3, *S. globosa* (CBS 120340); 4, *S. luriei* (CBS 937.72); 5, *S. mexicana* (CBS 120341); 6, *S. pallida* (CBS 302.73); 7, negative control. Amplicons were sized by comparison with bands of known size from the GeneRuler 100 bp Plus DNA Ladder (Thermo Fisher Scientific, USA). Direct visual detection of replicated DNA in RCA reactions with SYBR Green I (lower lanes). Tubes were imaged under ultraviolet light. Tube lanes: 1, *S. brasiliensis* (CBS 132990); 2, *S. schenckii* (CBS 359.36); 3, *S. globosa* (CBS 120340); 4, *S. luriei* (CBS 937.72); 5, *S. mexicana* (CBS 120341); 6, *S. pallida* (CBS 302.73); 7, negative control. Each padlock probe is specified.

RCA signal for the tested strains was easily visualized on 1.2% agarose gels. Interpretation of the RCA is straightforward and is based on a simple positive or negative result. Positive RCA generated a typical ladder-like pattern of fragments increasing in size, comprising the monomer and multimer repeats of the amplified product formed by single and multiple copying of the circularized padlock probe, while negative reactions had a clean background (Figure 3). RCA success was also determined by adding SYBR Green I dye after reactions; positive reactions fluoresced intensely, while negative reactions lacked fluorescence (Figure 3). The results from analysis with SYBR Green I dye were compatible with those obtained with electrophoresis (Figure 3). RCA-based identification completely matched (100%) identification via phylogenetic analysis (*kappa* 1.0; 95% confidence interval 1.0-1.0; Supplementary Table 1) and species-specific PCR (Rodrigues et al., 2015b) (*kappa* 1.0; 95% confidence interval 1.0-1.0; Supplementary Table 1).

The specificity of the identification assay was first determined for a subset of 40 *Sporothrix* isolates. Fifteen *S. schenckii* isolates belonging to different *CAL* haplotypes (Rodrigues et al., 2014d) were tested against species-specific padlock probes to evaluate the false-positive rate of the assay. No cross reactivity *in vitro* was apparent between closely related species, and all padlock probes yielded 100% specificity. Secondly, specificity of the identification was evaluated against other pathogenic or non-pathogenic fungi, and probes failed to replicate DNA from *Sporothrix brunneoviolacea, Sporothrix dimorphospora, Ophiostoma stenoceras, Paracoccidioides brasiliensis, Histoplasma capsulatum, Trichosporon asahii, Candida albicans*, and *Cryptococcus neoformans* (Supplementary Figure 1). Judging from *in vitro* results, our data supports the high specificity of the six padlock probes which was in good concordance with the results obtained via *in silico* predictions using BLASTn-search analyses.

**Figure 4:**
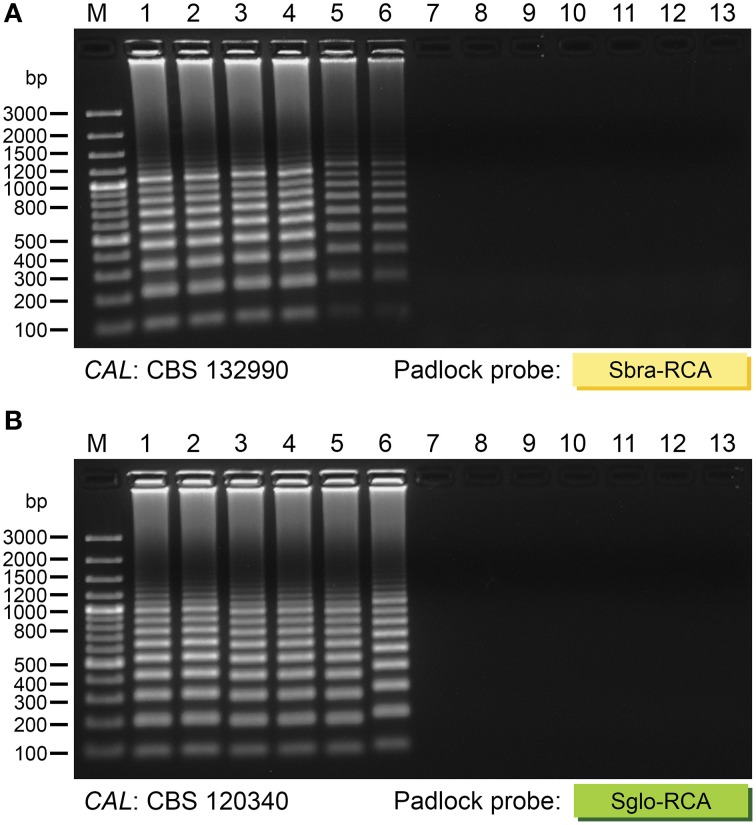
**Representative agarose gel electrophoresis of the analytical sensitivity of RCA**. Species-specific padlock probes were used for RCA of 10-fold dilutions of *CAL* amplicon from **(A)** *S. brasiliensis* (CBS 132990) and **(B)** *S. globosa* (CBS 120340). The in-gel detection limit was 3 × 10^6^ copies of *CAL* for all probes. Gel lanes: 1, 3 × 10^11^ copies; 2, 3 × 10^10^ copies; 3, 3 × 10^9^ copies; 4, 3 × 10^8^ copies; 5, 3 × 10^7^ copies; 6, 3 × 10^6^ copies; 7, 3 × 10^5^ copies; 8, 3 × 10^4^ copies; 9, 3 × 10^3^ copies; 10, 300 copies; 11, 30 copies; 12, 3 copies; 13, negative control. Marker (M): GeneRuler 100 bp Plus DNA Ladder (Thermo Fisher Scientific, USA).

The sensitivity of each probe was tested for the *CAL* sequence from a reference type strain of each species. *CAL* DNA was quantified and 10-fold diluted in ultra-pure water to achieve amplicon concentrations ranging from 3 × 10^11^ copies per tube to three copies per tube. RCA of these dilutions indicated excellent sensitivity, since all probes were detectable down to 3 × 10^6^ copies with equal reliability after electrophoresis (representative gels in Figure 4).

**Figure 5:**
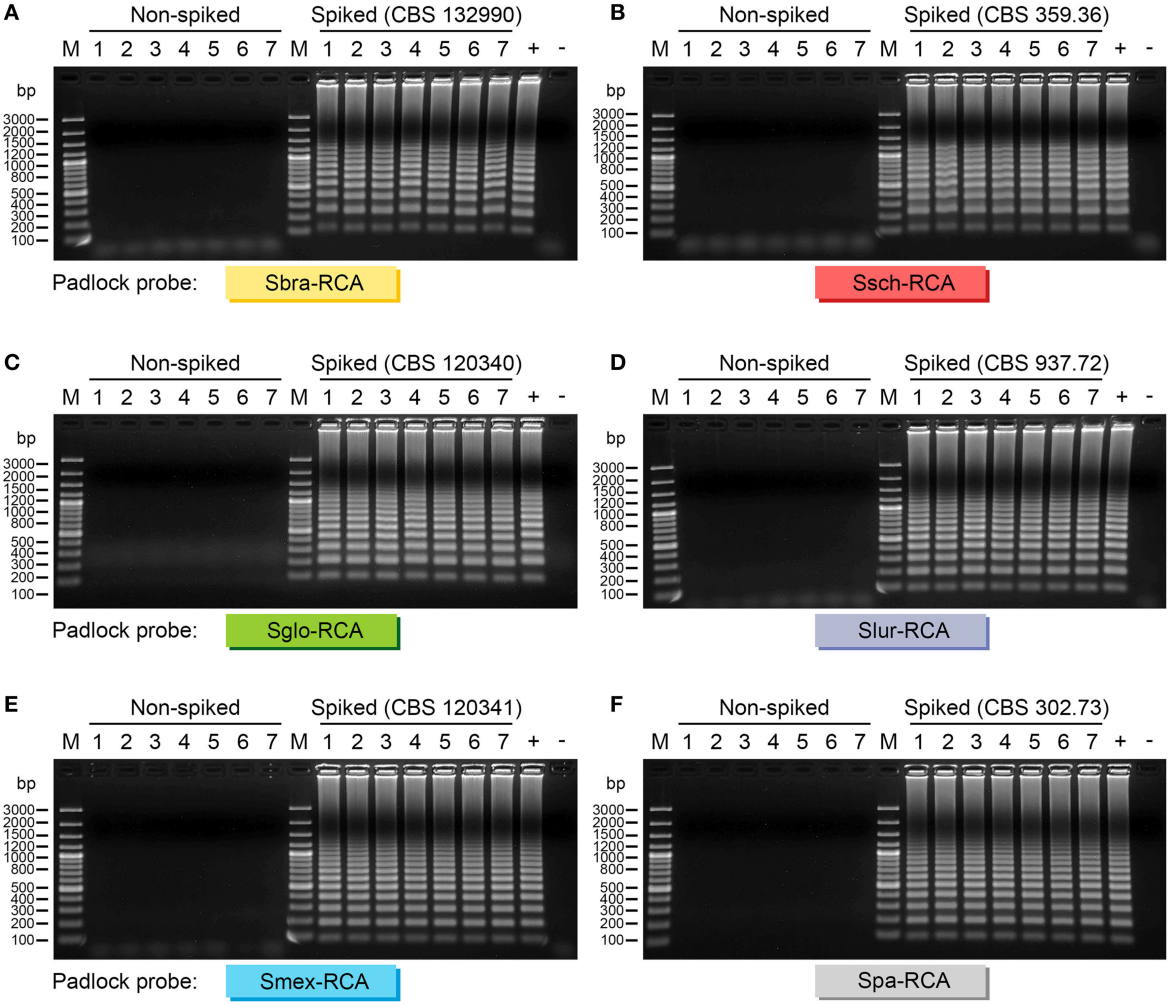
**Agarose gel electrophoresis of successful RCA amplification of members of ***Sporothrix schenckii***-***Ophiostoma stenoceras*** complex from a sample containing host/vegetable and fungal DNA**. **(A)** Sbra-RCA padlock probe; **(B)** Ssch-RCA padlock probe; **(C)** Sglo-RCA padlock probe; **(D)** Slur-RCA padlock probe; **(E)** Smex-RCA padlock probe; and **(F)** Spa-RCA padlock probe. Gel lanes 1–7 on the left correspond to non-spiked samples (negative control): 1, soil; 2, *Sphagnum* moss; 3, *Rose* spp.; 4, BALB/c spleen; 5, BALB/c lungs; 6, BALB/c tail; 7, BALB/c feces. Gel lanes 1–7 on the right correspond to spiked samples (species as indicated): 1, spiked soil; 2, spiked *Sphagnum* moss; 3, spiked *Rose* spp.; 4, spiked BALB/c spleen; 5, spiked BALB/c lungs; 6, spiked BALB/c tail; 7, spiked BALB/c feces. +, positive control using DNA from culture; -, negative control (blank). Amplicons were sized by comparison with bands of known size from the GeneRuler 100 bp Plus DNA Ladder (M) (Thermo Fisher Scientific, USA). The results show an efficient replication of samples spiked with pathogen DNA. On the other hand, RCA failed to replicate the DNA originated solely from environmental or clinical samples (non-spiked), which remained negative. It can thus be assumed that the padlock probes can selectively replicate pathogen DNA, confirming the high specificity of our RCA-based assay.

Furthermore, we evaluated if padlock probes could selectively detect *Sporothrix* DNA from complex environmental and animal samples. Spiked samples were used to determine whether DNA detection was affected by the presence of different mixtures of the biological samples, what is essential for validating and assessing the accuracy of RCA in detecting pathogen-specific DNA. Judging from both environmental and clinical samples, we successfully detected positive RCA signals as a typical ladder-like pattern from soil, *Sphagnum* moss, *Rose* spp., spleen, lungs, tail, and feces of mouse spiked with *Sporothrix* DNA. No false positives were detected in samples from the control group (non-spiked samples), which remained as a clean background. It can thus be assumed that the padlock probes can selectively replicate pathogen DNA, confirming the high specificity of our RCA-based assay as well as its potential applicability to detection studies (Figure 5).

## Discussion

The search for techniques that avoid variability and lower costs for identifying pathogenic *Sporothrix* species motivated us to develop and evaluate an RCA-based assay for this application. RCA is a powerful method for the molecular characterization of medically important fungi (Najafzadeh et al., 2011, 2013; Sun et al., 2011; Davari et al., 2012; Lackner et al., 2012; Feng et al., 2013; Hamzehei et al., 2013). Here, we report the successful use of six species-specific padlock probes for fast, robust, and accurate diagnosis of sporotrichosis.

We redesigned the primer pair targeting the *CAL* gene in order to increase the sensitivity and specificity of PCR amplification. Degenerate primers that target *CAL* (CL1 and CL2A, O'Donnell et al., 2000) are associated with varying sensitivity and success of amplification depending on the species assayed (Zhang et al., 2015). The new primers developed here (*CAL*-Fw and *CAL*-Rv) are directly applicable to molecular phylogeny studies of *Sporothrix*/*Ophiostoma* species. Successful amplification was confirmed with 96 DNA samples mainly derived from *Sporothrix* isolates recovered from clinical cases in Latin America and other regions of the world (Table 1). The species successfully amplified by PCR included *S. brasiliensis, S. schenckii s. str., S. globosa, S. luriei, S. mexicana, S. pallida, S. chilensis, S. brunneoviolaceae*, and *S. dimorphospora*. Although the use of degenerate primers CL1 and CL2A increases the flexibility of PCR, low amounts of amplicon are usually recovered for *S. luriei* (Rodrigues et al., 2014b), *S. schenckii, S. globosa*, and especially for isolates embedded in the environmental clade (Zhang et al., 2015). Therefore, we propose a new primer combination (*CAL*-Fw and *CAL*-Rv) that covers the region amplified using the CL1 and CL2A primers (exons 3–5) but generates slightly longer amplicons and higher-quality sequences.

We also designed and tested padlock probes to identify polymorphic sequences produced by PCR of *CAL*. After ligation, we removed nucleotides from single-stranded DNA (non-circularized padlock probes and excess primer) with Exo I and Exo III. Although this step has been regarded as optional (Sun et al., 2011; Davari et al., 2012; Najafzadeh et al., 2013; Dolatabadi et al., 2014), we included it in order to avoid non-specific amplification during RCA. It is important to note that, similar to the results obtained by Ahmed et al. (2014) for agents of eumycetoma, exonucleolysis during *Sporothrix* identification may generate low RCA-positive signal on electrophoresis or with fluorescence (Supplementary Figure 2). In the current investigation, RCA driven by *Bst* DNA polymerase successfully replicated *Sporothrix* circularized padlock probes with high specificity. Gel-free systems that use intercalating fluorescent dye to visualize amplified product are particularly interesting due to the shorter amount of time necessary for accurate results. SYBR Green I dye is a quick and inexpensive reagent for assessing RCA without the routine use of electrophoresis, which is especially attractive for investigations in remote areas where sporotrichosis is endemic but where equipment for molecular diagnosis is lacking.

Here, *Sporothrix* RCA was sensitive. We observed a typical ladder-like pattern on gel electrophoresis with as few as 3 × 10^6^ copies of *CAL* amplicon per tube. This sensitivity is similar to previous results reported for *Scedosporium* (Lackner et al., 2012), eumycetoma-causing agents (Ahmed et al., 2014), and the mucorales (Dolatabadi et al., 2014).

We determined that identification via *Sporothrix*-specific RCA was 100% in agreement with phylogenetic analysis (Rodrigues et al., 2014d), analysis with *CAL*-RFLPs (Rodrigues et al., 2014b), and the application of species-specific primers (Rodrigues et al., 2015b) (Supplementary Table 1). The optimal method for identifying *Sporothrix* may depend on sample origin (soil, plant debris, animal biopsies, or isolated culture), DNA quality, and the price and sensitivity of the molecular assay. RCA, PCR-RFLP (Rodrigues et al., 2014b), and species-specific primers (Rodrigues et al., 2015b) reliably identify *Sporothrix* pathogenic agents down to species level. However, *CAL*-RFLP (Rodrigues et al., 2014b) is not recommended for detecting *Sporothrix* in complex mixtures of DNA such as biopsy material or in crude environmental samples such as soil and decaying wood. On the other hand, RCA and species-specific primers (Rodrigues et al., 2015b) may perform well in these situations, with similar sensitivity, depending on sample preparation.

We demonstrated for the first time that padlock probes are useful for tracking *Sporothrix* in ecological studies, e.g., for monitoring the presence of *Sporothrix* DNA from soil and plant debris, the most common environmental sources during outbreaks. Isolation of *Sporothrix* spp. from soil samples is reported in endemic areas (Mehta et al., 2007; Criseo and Romeo, 2010; Montenegro et al., 2014; Govender et al., 2015), however, only a few studies performed molecular characterization of environmental isolates down to species level. Notwithstanding, the distributions of medically-relevant *Sporothrix* in soil and the conditions that increase its occurrence have been the subject of various hypothesis (Zhang et al., 2015). *Sporothrix* is embedded in the Ophiostomatales, an order that comprises many ecological niches. These microorganisms are adapted for dispersal by insects (Coleoptera: Scolytinae), are associated with the *Protea, Rosa* spp., *Sphagnum* moss or are widely distributed in the soil (De Beer and Wingfield, 2013). In this scenario, the RCA-based assay developed here is an important tool for helping to detect *Sporothrix* DNA directly in complex environmental samples, as a reliable alternative to DNA sequencing, expanding our knowledge on the distribution of these microorganisms in nature, especially in endemic regions.

Sporotrichosis primarily affects warm-blooded animals, particularly humans and cats. The disease is emerging as a global threat, with high incidences in somewhat warmer regions, but it still bears with it the problem of lack of early diagnosis associated with correct species identification. To evaluate the capacity of padlock probes detecting *Sporothrix* DNA from clinical samples of warm-blooded animals, we used DNA from BALB/c mice spiked with known *Sporothrix* species. All six padlock probes showed a high degree of specificity that makes them useful for detecting medically-relevant *Sporothrix* DNA sequences even in the presence of host DNA. Our data also shows that within host samples, the presence of *Sporothrix* DNA can also be explored in feces, as demonstrated earlier by species-specific PCR (Rodrigues et al., 2015b). Indeed, feces from infected animals may be valuable samples for exploring the ecological and epidemiological features of sporotrichosis, especially because it can increase the number of foci in the environment (Schubach et al., 2003; Montenegro et al., 2014). Our RCA assay may also be useful when large-scale screening of *Sporothrix* spp. is required, such as in Brazil, China, and South Africa where sporotrichosis is still endemic and remains a major public health problem (Zhou et al., 2014; Govender et al., 2015; Zhang et al., 2015).

Unambiguous identification of human-pathogenic *Sporothrix* spp. based on phenotypic methods is rather challenging due to overlapping morphologies among closely related species. In this scenario, these difficulties delay the response to an outbreak and/or its epidemiological surveillance. To overcome this problem, we first demonstrated *in silico* that padlock probes are particularly amenable to discriminate specific polymorphisms in the *CAL* sequence that can be used to speciate all agents embedded in the *S*. *schenckii* complex. Second, we validate *in vitro* the feasibility of the RCA method for sensitive and sequence-specific detection of *Sporothrix* DNA derive from pure cultures. Third, as a proof of concept, we used this new RCA-based approach to successfully detect *Sporothrix* DNA from a complex mixture containing host/vegetable nucleic acids. The technique has several advantages, including high sensitivity, high specificity, fast, easy to perform, simple to interpret, and the lack of a need for special instrumentation; which is especially desirable during epidemics, when thousands of samples must be accurately identified. A single RCA assay (including mix preparation, ligation and amplification) takes less than 3 h. These findings are likely to improve identification, diagnosis, to guide patient treatment, to improve clinical outcomes, and to tackle the spread of future outbreaks. Due to these robustness and simplicity characteristics, RCA may also be useful in low-income regions where human and animal sporotrichosis is endemic and epidemic.

### Conflict of interest statement

The authors declare that the research was conducted in the absence of any commercial or financial relationships that could be construed as a potential conflict of interest.
