# Supplementary material for: Rapid Identification of Emerging Human-Pathogenic Sporothrix Species with Rolling Circle Amplification
Source: Front Microbiol. 2015 Dec 8;6:1385. doi: 10.3389/fmicb.2015.01385 (PMC4672047; doi:10.3389/fmicb.2015.01385)
Supplement: Supplementary file 1 [file Image1.PDF]

Supplementary Material

Rapid Identification of Emerging Human-pathogenic *Sporothrix* Species with Rolling Circle Amplification

Anderson Messias Rodrigues[1,]*, Mohammad Javad Najafzadeh[2], G. Sybren de Hoog[3], Zoilo Pires de Camargo[1,]*

*** Correspondence:** amrodrigues.amr@gmail.com (AMR) and zpcamargo1@gmail.com (ZPdC).

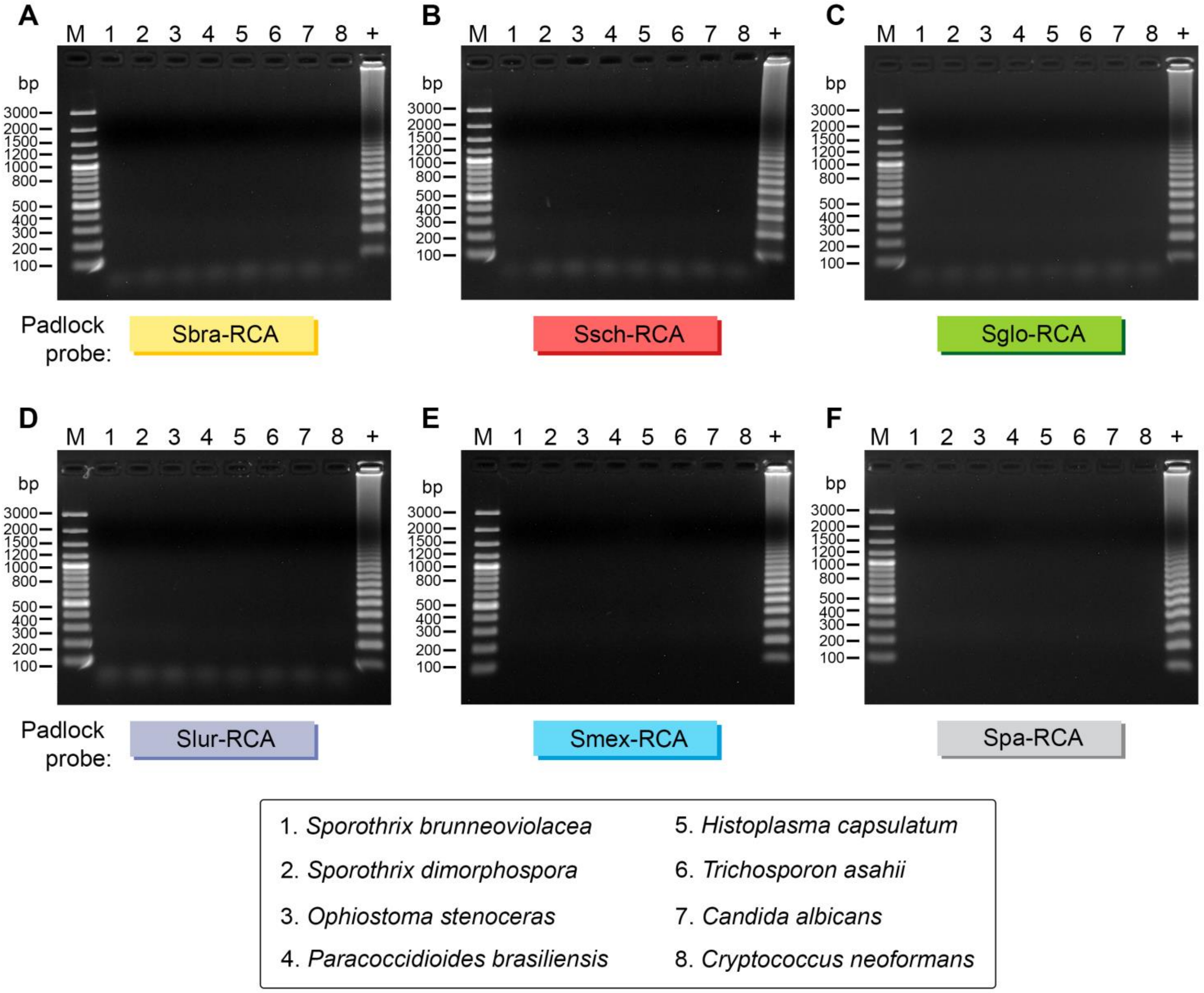

Supplementary Figure 1. Agarose gel electrophoresis of RCA showing absence of amplification for *Sporothrix brunneoviolacea* (CBS 124561; lane 1), *Sporothrix dimorphospora* (CBS 125442; lane 2), *Ophiostoma stenoceras* (C8213, lane 3), *Paracoccidioides brasiliensis* (Pb18, lane 4), *Histoplasma capsulatum* (832, lane 5), *Trichosporon asahii* (CBS 2530, lane 6), *Candida albicans* (ATCC 10231,

lane 7) and *Cryptococcus neoformans* (CBS 10079, lane 8) using (A) Sbra-RCA padlock probe (positive control: CBS 132990); (B) Ssch-RCA padlock probe (positive control: CBS 359.36); (C) Sglo-RCA padlock probe (positive control: CBS 120340); (D) Slur-RCA padlock probe (positive control: CBS 937.72); (E) Smex-RCA padlock probe (positive control: CBS 120341); and (F) Spa-RCA padlock probe (positive control: CBS 302.73). Type strains were used as positive controls (+). Amplicons were sized by comparison with bands of known size from the GeneRuler 100 bp Plus DNA Ladder (Thermo Fisher Scientific, USA).
