# Supplementary material for: Rapid Identification of Emerging Human-Pathogenic Sporothrix Species with Rolling Circle Amplification
Source: Front Microbiol. 2015 Dec 8;6:1385. doi: 10.3389/fmicb.2015.01385 (PMC4672047; doi:10.3389/fmicb.2015.01385)
Supplement: Supplementary file 2 [file Image2.PDF]

frontiers

Supplementary Material

Rapid Identification of Emerging Human-pathogenic *Sporothrix* Species with Rolling Circle Amplification

Anderson Messias Rodrigues[1,]*, Mohammad Javad Najafzadeh[2], G. Sybren de Hoog[3], Zoilo Pires de Camargo[1,]*

*** Correspondence:** amrodrigues.amr@gmail.com (AMR) and zpcamargo1@gmail.com (ZPdC).

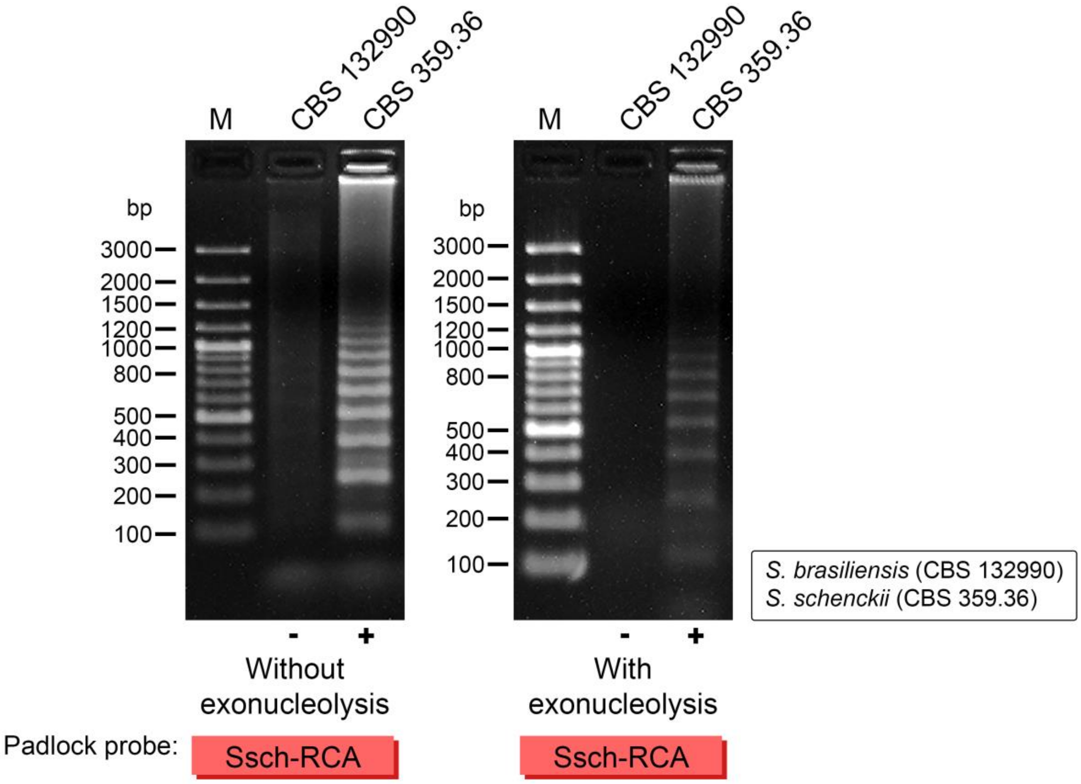

Supplementary Figure 2. Agarose gel electrophoresis of successful RCA of Ssch-RCA padlock probe and *CAL* amplicon of *S. schenckii* isolate CBS 359.36 and absence of amplification of *S. brasiliensis* isolate CBS 132990 without (left) and with (right) exonucleolysis treatment. After ligation, nucleotides from single-stranded DNA (non-circularized padlock probes and excess primer) can be removed enzymatically with Exo I and Exo III treatment. Nevertheless, this step has been regarded as optional by several authors (Please, see Sun et al., 2011;Davari et al., 2012;Najafzadeh et al., 2013;Dolatabadi et al., 2014). Note that a weak background can be observed for *S. brasiliensis* without exonucleolysis treatment. However, it should not be regarded as a false positive, because it lacked a typical ladder-like pattern of fragments increasing in size. On the other hand, treatment with Exo I and Exo III removed the weak background but it may generate weaker positive reactions

(compared to non-treated probes). Since this step (exonucleolysis) is optional, RCA reactions may be interpreted as positive only in the presence a typical ladder-like pattern of fragments increasing in size (as shown for CBS 359.36 in both gels).

Sun, J., Najafzadeh, M.J., Zhang, J., Vicente, V.A., Xi, L., and De Hoog, G.S. (2011). Molecular identification of *Penicillium marneffei* using rolling circle amplification. Mycoses 54, e751-759.

Davari, M., Van Diepeningen, A.D., Babai-Ahari, A., Arzanlou, M., Najafzadeh, M.J., Van Der Lee, T.A., and De Hoog, G.S. (2012). Rapid identification of *Fusarium graminearum* species complex using Rolling Circle Amplification (RCA). J Microbiol Methods 89, 63-70.

Najafzadeh, M.J., Dolatabadi, S., Saradeghi Keisari, M., Naseri, A., Feng, P., and De Hoog, G.S. (2013). Detection and identification of opportunistic *Exophiala* species using the rolling circle amplification of ribosomal internal transcribed spacers. J Microbiol Methods 94, 338-342.

Dolatabadi, S., Najafzadeh, M.J., and De Hoog, G.S. (2014). Rapid screening for human-pathogenic Mucorales using rolling circle amplification. Mycoses 57, 67-72.
