# Supplementary material for: Rapid Identification of Emerging Human-Pathogenic Sporothrix Species with Rolling Circle Amplification
Source: Front Microbiol. 2015 Dec 8;6:1385. doi: 10.3389/fmicb.2015.01385 (PMC4672047; doi:10.3389/fmicb.2015.01385)
Supplement: Supplementary file 3 [file Table1.PDF]

Supplementary Material

Rapid Identification of Emerging Human-pathogenic *Sporothrix* Species with Rolling Circle Amplification

Anderson Messias Rodrigues[1,*], Mohammad Javad Najafzadeh[2], G. Sybren de Hoog[3], Zoilo Pires de Camargo[1,*]

*** Correspondence:** amrodrigues.amr@gmail.com (AMR) and zpcamargo1@gmail.com (ZPdC).

Supplementary Table 1. Strains and species identification using phylogeny, species-specific PCR, *CAL*-RFLP, and rolling circle amplification.

Isolate code	CBS code	Species Identification			
		***CAL*+ITS Phylogeny[1]**	**Species-specific PCR[2]**	**PCR-RFLP of *CAL*[3]**	**RCA[This study]**
Ss05	CBS 132985	*S. brasiliensis*	*S. brasiliensis*	*S. brasiliensis*	*S. brasiliensis*
Ss07	CBS 132986	*S. brasiliensis*	*S. brasiliensis*	*S. brasiliensis*	*S. brasiliensis*
Ss12	-	*S. brasiliensis*	*S. brasiliensis*	*S. brasiliensis*	*S. brasiliensis*
Ss14	-	*S. brasiliensis*	*S. brasiliensis*	*S. brasiliensis*	*S. brasiliensis*
Ss33	-	*S. brasiliensis*	*S. brasiliensis*	*S. brasiliensis*	*S. brasiliensis*
Ss34	-	*S. brasiliensis*	*S. brasiliensis*	*S. brasiliensis*	*S. brasiliensis*
Ss37	-	*S. brasiliensis*	*S. brasiliensis*	*S. brasiliensis*	*S. brasiliensis*
Ss38	-	*S. brasiliensis*	*S. brasiliensis*	*S. brasiliensis*	*S. brasiliensis*
Ss43	-	*S. brasiliensis*	*S. brasiliensis*	*S. brasiliensis*	*S. brasiliensis*
Ss44	-	*S. brasiliensis*	*S. brasiliensis*	*S. brasiliensis*	*S. brasiliensis*
Ss52	-	*S. brasiliensis*	*S. brasiliensis*	*S. brasiliensis*	*S. brasiliensis*
Ss54	CBS 132990	*S. brasiliensis*	*S. brasiliensis*	*S. brasiliensis*	*S. brasiliensis*
Ss55	-	*S. brasiliensis*	*S. brasiliensis*	*S. brasiliensis*	*S. brasiliensis*
Ss56	-	*S. brasiliensis*	*S. brasiliensis*	*S. brasiliensis*	*S. brasiliensis*
Ss57	-	*S. brasiliensis*	*S. brasiliensis*	*S. brasiliensis*	*S. brasiliensis*
Ss62	CBS 132991	*S. brasiliensis*	*S. brasiliensis*	*S. brasiliensis*	*S. brasiliensis*
Ss69	-	*S. brasiliensis*	*S. brasiliensis*	*S. brasiliensis*	*S. brasiliensis*
Ss82	CBS 132992	*S. brasiliensis*	*S. brasiliensis*	*S. brasiliensis*	*S. brasiliensis*
Ss87	CBS 132993	*S. brasiliensis*	*S. brasiliensis*	*S. brasiliensis*	*S. brasiliensis*
Ss99	-	*S. brasiliensis*	*S. brasiliensis*	*S. brasiliensis*	*S. brasiliensis*
Ss104	-	*S. brasiliensis*	*S. brasiliensis*	*S. brasiliensis*	*S. brasiliensis*
Ss128	-	*S. brasiliensis*	*S. brasiliensis*	*S. brasiliensis*	*S. brasiliensis*
Ss265	CBS 133020	*S. brasiliensis*	*S. brasiliensis*	*S. brasiliensis*	*S. brasiliensis*
ATCC 4823[G]	CBS 132021	*S. brasiliensis*	*S. brasiliensis*	*S. brasiliensis*	*S. brasiliensis*
IPEC16490[T]	CBS 120339 [T]	*S. brasiliensis*	*S. brasiliensis*	*S. brasiliensis*	*S. brasiliensis*

Isolate code	CBS code	Species Identification			
		CAL+ITS Phylogeny[1]	Species-specific PCR[2]	PCR-RFLP of *CAL*[3]	RCA[This study]
Ss06	CBS 132922	*S. globosa*	*S. globosa*	*S. globosa*	*S. globosa*
Ss41	CBS 132923	*S. globosa*	*S. globosa*	*S. globosa*	*S. globosa*
Ss49	CBS 132924	*S. globosa*	*S. globosa*	*S. globosa*	*S. globosa*
Ss236	CBS 132925	*S. globosa*	*S. globosa*	*S. globosa*	*S. globosa*
FMR 8600[T]	CBS 120340	*S. globosa*	*S. globosa*	*S. globosa*	*S. globosa*
FMR 9280[T]	CBS 937.72	*S. luriei*	-	*S. luriei*	*S. luriei*
Ss132	CBS 132927	*S. mexicana*	*S. mexicana*	-	*S. mexicana*
Ss133	CBS 132928	*S. mexicana*	*S. mexicana*	-	*S. mexicana*
FMR 9107	CBS 120342	*S. mexicana*	*S. mexicana*	-	*S. mexicana*
FMR 9108[T]	CBS 120341	*S. mexicana*	*S. mexicana*	-	*S. mexicana*
FMR 8939[T]	CBS 302.73	*S. pallida*	*S. pallida*	-	*S. pallida*
Ss327	-	*S. pallida*	*S. pallida*	-	*S. pallida*
CBS 111110	CBS 111110	*S. pallida*	*S. pallida*	-	*S. pallida*
Ss03	CBS 132963	*S. schenckii*	*S. schenckii*	*S. schenckii*	*S. schenckii*
Ss04	-	*S. schenckii*	*S. schenckii*	*S. schenckii*	*S. schenckii*
Ss13	-	*S. schenckii*	*S. schenckii*	*S. schenckii*	*S. schenckii*
Ss15	-	*S. schenckii*	*S. schenckii*	*S. schenckii*	*S. schenckii*
Ss16	-	*S. schenckii*	*S. schenckii*	*S. schenckii*	*S. schenckii*
Ss17	-	*S. schenckii*	*S. schenckii*	*S. schenckii*	*S. schenckii*
Ss19	-	*S. schenckii*	*S. schenckii*	*S. schenckii*	*S. schenckii*
Ss22	CBS 132964	*S. schenckii*	*S. schenckii*	*S. schenckii*	*S. schenckii*
Ss36	-	*S. schenckii*	*S. schenckii*	*S. schenckii*	*S. schenckii*
Ss39	-	*S. schenckii*	*S. schenckii*	*S. schenckii*	*S. schenckii*
Ss40	-	*S. schenckii*	*S. schenckii*	*S. schenckii*	*S. schenckii*
Ss42	CBS 132966	*S. schenckii*	*S. schenckii*	*S. schenckii*	*S. schenckii*
Ss45	-	*S. schenckii*	*S. schenckii*	*S. schenckii*	*S. schenckii*
Ss46	-	*S. schenckii*	*S. schenckii*	*S. schenckii*	*S. schenckii*
Ss47	-	*S. schenckii*	*S. schenckii*	*S. schenckii*	*S. schenckii*
Ss48	-	*S. schenckii*	*S. schenckii*	*S. schenckii*	*S. schenckii*
Ss58	-	*S. schenckii*	*S. schenckii*	*S. schenckii*	*S. schenckii*
Ss59	-	*S. schenckii*	*S. schenckii*	*S. schenckii*	*S. schenckii*
Ss61	-	*S. schenckii*	*S. schenckii*	*S. schenckii*	*S. schenckii*
Ss63	CBS 132968	*S. schenckii*	*S. schenckii*	*S. schenckii*	*S. schenckii*
Ss64	-	*S. schenckii*	*S. schenckii*	*S. schenckii*	*S. schenckii*
Ss73	-	*S. schenckii*	*S. schenckii*	*S. schenckii*	*S. schenckii*
Ss75	-	*S. schenckii*	*S. schenckii*	*S. schenckii*	*S. schenckii*
Ss78	-	*S. schenckii*	*S. schenckii*	*S. schenckii*	*S. schenckii*
Ss80	CBS 132969	*S. schenckii*	*S. schenckii*	*S. schenckii*	*S. schenckii*
Ss102	CBS 132970	*S. schenckii*	*S. schenckii*	*S. schenckii*	*S. schenckii*
Ss105	-	*S. schenckii*	*S. schenckii*	*S. schenckii*	*S. schenckii*
Ss107	-	*S. schenckii*	*S. schenckii*	*S. schenckii*	*S. schenckii*
Ss109	-	*S. schenckii*	*S. schenckii*	*S. schenckii*	*S. schenckii*
Ss110	-	*S. schenckii*	*S. schenckii*	*S. schenckii*	*S. schenckii*
Ss113	CBS 132972	*S. schenckii*	*S. schenckii*	*S. schenckii*	*S. schenckii*

Isolate code	CBS code	Species Identification			
		CAL+ITS Phylogeny[1]	Species-specific PCR[2]	PCR-RFLP of *CAL*[3]	RCA[This study]
Ss116	-	*S. schenckii*	*S. schenckii*	*S. schenckii*	*S. schenckii*
Ss118	CBS 132974	*S. schenckii*	*S. schenckii*	*S. schenckii*	*S. schenckii*
Ss119	-	*S. schenckii*	*S. schenckii*	*S. schenckii*	*S. schenckii*
Ss122	-	*S. schenckii*	*S. schenckii*	*S. schenckii*	*S. schenckii*
Ss123	-	*S. schenckii*	*S. schenckii*	*S. schenckii*	*S. schenckii*
Ss124	-	*S. schenckii*	*S. schenckii*	*S. schenckii*	*S. schenckii*
Ss126	-	*S. schenckii*	*S. schenckii*	*S. schenckii*	*S. schenckii*
Ss129	-	*S. schenckii*	*S. schenckii*	*S. schenckii*	*S. schenckii*
Ss130	-	*S. schenckii*	*S. schenckii*	*S. schenckii*	*S. schenckii*
Ss136	-	*S. schenckii*	*S. schenckii*	*S. schenckii*	*S. schenckii*
Ss137	-	*S. schenckii*	*S. schenckii*	*S. schenckii*	*S. schenckii*
Ss138	-	*S. schenckii*	*S. schenckii*	*S. schenckii*	*S. schenckii*
Ss140	-	*S. schenckii*	*S. schenckii*	*S. schenckii*	*S. schenckii*
Ss141	CBS 132975	*S. schenckii*	*S. schenckii*	*S. schenckii*	*S. schenckii*
Ss143	-	*S. schenckii*	*S. schenckii*	*S. schenckii*	*S. schenckii*
Ss144	-	*S. schenckii*	*S. schenckii*	*S. schenckii*	*S. schenckii*
Ss158	-	*S. schenckii*	*S. schenckii*	*S. schenckii*	*S. schenckii*
Ss190	-	*S. schenckii*	*S. schenckii*	*S. schenckii*	*S. schenckii*
Ss240	-	*S. schenckii*	*S. schenckii*	*S. schenckii*	*S. schenckii*
Ss159	CBS 132976	*S. schenckii*	*S. schenckii*	*S. schenckii*	*S. schenckii*
Ss160	-	*S. schenckii*	*S. schenckii*	*S. schenckii*	*S. schenckii*
Ss161	-	*S. schenckii*	*S. schenckii*	*S. schenckii*	*S. schenckii*
Ss162	CBS 132977	*S. schenckii*	*S. schenckii*	*S. schenckii*	*S. schenckii*
Ss163	-	*S. schenckii*	*S. schenckii*	*S. schenckii*	*S. schenckii*
Ss164	-	*S. schenckii*	*S. schenckii*	*S. schenckii*	*S. schenckii*
ATCC 4821[G]	CBS 132984	*S. schenckii*	*S. schenckii*	*S. schenckii*	*S. schenckii*
CBS 359.36[T]	CBS 359.36	*S. schenckii*	*S. schenckii*	*S. schenckii*	*S. schenckii*

IPEC, Instituto de Pesquisa Clínica Evandro Chagas, Fiocruz, Brazil; FMR, Facultat de Medicina i Ciències de la Salut, Reus, Spain; CBS, Centraalbureau voor Schimmelcultures, Utrecht, The Netherlands; ATCC: American Type Culture Collection, Manassas, USA; NK, not known; [T], type strain; [G], genome. All "Ss" strains belong to the culture collection of Federal University of SP (UNIFESP).

1. Rodrigues AM, de Hoog GS, Zhang Y, Camargo ZP, 2014. Emerging sporotrichosis is driven by clonal and recombinant *Sporothrix* species. Emerg Microbes Infect 3: e32.
2. Rodrigues AM, de Hoog GS, Camargo ZP, 2015. Molecular diagnosis of pathogenic *Sporothrix* species. PLoS Negl Trop Dis: 10.1371/journal.pntd.0004190.
3. Rodrigues AM, de Hoog GS, Camargo ZP, 2014. Genotyping species of the *Sporothrix schenckii* complex by PCR-RFLP of calmodulin. Diagn Microbiol Infect Dis 78: 383–387.
